# Machine Retrograde Perfusion of Deceased Donor Kidneys: A Prospective Study

**DOI:** 10.3389/fmed.2021.785953

**Published:** 2021-12-17

**Authors:** Jun Zeng, ZiHao Jia, Tao Lin, TuRun Song

**Affiliations:** ^1^Department of Urology, Institute of Urology, West China Hospital, Sichuan University, Chengdu, China; ^2^Organ Transplantation Center, West China Hospital, Sichuan University, Chengdu, China

**Keywords:** deceased donor kidneys, retrograde perfusion, kidney transplantation, LifePort, organ recovery

## Abstract

**Objective:** To maximize the utilization of potential kidneys, improving perfusion and preservation techniques is necessary.

**Methods:** We investigated the safety and efficacy of retrograde machine perfusion of kidneys from deceased donors. A total of 30 kidneys were included and all the grafts were preserved in the Kidney Transporter machines. A total of 15 kidneys that received retrograde perfusion (RP) were selected as the RP group (*n* = 15) and their counterparts received standard antegrade perfusion (AP) as the control group (*n* = 15).

**Results:** All the recipients were followed up for 6 months. Renal resistance in the RP group remained stable during the perfusion. There was no primary nonfunction. No difference in the incidence of delayed graft function was found in both groups (3 in RP vs. 2 in AP, *p* = 0.62). The RP group had lower serum creatinine (RP vs. AP, 102.20 vs. 138.67, *p* = 0.05) and blood urea nitrogen (RP vs. AP, 6.44 vs. 8.71, *p* = 0.05) than that in the AP group at 6 months. Both the groups had comparable estimated glomerular filtration rate and cystatin C within 6 months.

**Conclusion:** This novel technique may be an effective and safe alternative for kidney preservation.

## Introduction

Kidney transplantation is the treatment of option for end-stage renal disease (ESRD) (1). However, there is still a major discrepancy between the kidney available for transplantation and the actual demand, resulting in an increasing number on the waiting list (2). Efforts should be made to utilize any potential kidney grafts. Besides living donation, kidneys from deceased, old, and “marginal or expanded” donors are the essential source to expand the donor pool (3). However, organs from these donors are associated with higher rates of being discarded, especially when they are not well perfused. Therefore, novel preservation techniques should be adopted and increase the utilization of these organs. Hypothermic machine perfusion (HMP) answers this call and mounting evidence has indicated that HMP had reduced delayed graft function (DGF), better recovery, and kidney function compared with static cold storage (SCS) (4).

The current standard practice of HMP involves perfusion of cold preservation solution into the kidney via a cannula connected to the renal artery. In procurement, we may encounter multiple renal arteries, artery spasm, or intraoperative damage to the arteries. In these circumstances, the conventional antegrade perfusion (AP) is not a proper technique because it might lead to unsatisfied kidney perfusion and inferior clinical outcomes after transplantation (5–8), even organ discarded. Previous studies have indicated that retrograde perfusion (RP) through the inferior vena cava in some cardiothoracic surgery can protect abdominal organs and kidneys (9–11). Han et al. even showed the feasibility and efficacy of RP in kidney graft from rabbits, sheep, and pigs (11, 12). Inspired by the aforementioned findings, we utilized HMP with RP technique to perfuse kidneys from deceased donors. In this study, we reported the short-term results of these novel techniques.

## Patients and Methods

### Patients

This study is a prospective observation of kidney transplants from deceased donors in West China Hospital, Sichuan University (ClinicalTrials.gov ID: NCT04569682). The institutional review board approved the study protocol and authorized data collection and we obtained the consensus from all the participants. All the kidney grafts were procured from donation after brain death (DBD) between January 1, 2020, and August 1, 2020, with conventional perfusion through a lower segment of the abdominal aorta. When the procurement was completed, kidneys were immediately placed in the ice water for vascular clip on the table preparation. All the right renal veins were lengthening with inferior vena cava for surgical convenience. After that, all the kidneys were perfused with HMP in the LifePort Kidney Transporter machines (Organ Recovery Systems Incorporation, Itasca, Illinois, USA) until operation (13). A total of 30 kidneys were randomized to receive AP and RP. Consequently, their recipients were selected into the AP group (*n* = 15) and the RP group (*n* = 15). Demographic data of both the donors and recipients, human leukocyte antigen (HLA) mismatch, warm ischemia time (WIT), cold ischemia time (CIT), perfusion time (PT), urine output, DGF (defined as requiring dialysis in the first week), serum creatinine (Scr), blood urea nitrogen (BUN), estimated glomerular filtration rate (eGFR), and cystatin C (CysC) at postoperative day (POD) 1, 2, 3, 4, 7, 14, 21, 30, 60, 90, 120, 150, and 180 were collected. Ultrasonic arterial resistance 1 week after the operation was compared as well. No executed prisoners were used as donors and participants were neither paid nor coerced in this study.

### Hypothermic Machine Perfusion

**Figure 1:**
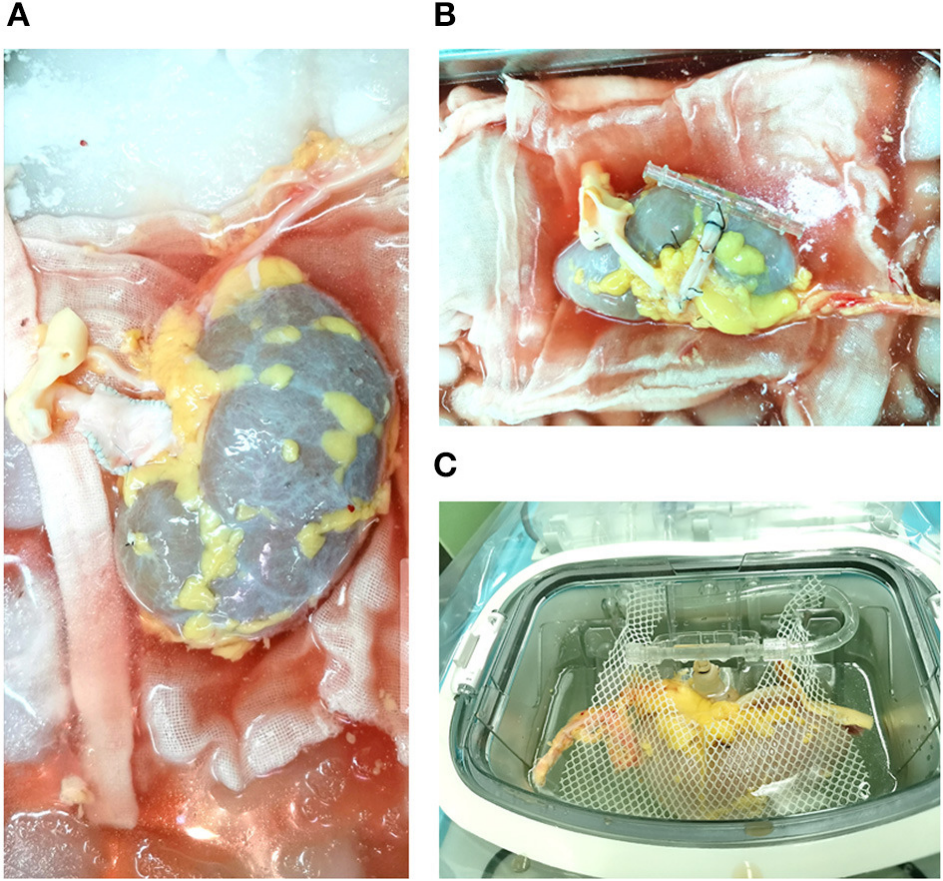
Donor kidney perfused by the retrograde technique. **(A)** Lengthening the right renal vein for kidney graft; **(B)** A catheter was inserted into the renal vein; **(C)** kidney graft was perfused by the retrograde technique in the LifePort Kidney Transporter machines.

All the kidneys were preserved by the LifePort Kidney Transporter machines (Organ Recovery Systems Incorporation, Itasca, Illinois, USA). The technique for RP was described as follows: a catheter was inserted into the renal vein and the RP was performed with a pulsatile flow of kidney preservation solution-1 (14) at 1 to 8°C (Figure 1). The initial perfusion pressure was set at 15 mm Hg. If the perfusion went well, perfusion pressure was gradually reduced to 12 mm Hg with 1 mm Hg lower every 10 min. Otherwise, the pressure was maintained at 15 mm Hg. The initial perfusion pressure was set at 30 mm Hg in the AP group and all the kidneys were preserved with machine perfusion until transplantation. The machine perfusion time, pressure, flow, and resistance index were recorded and analyzed.

### Statistical Analysis

Continuous data were represented as the mean ± SD and assessed by the Student's *t-*test and the Mann–Whitney U test to evaluate the differences between the AP and the RP groups. Categorical data were expressed as frequency and analyzed with the chi-squared test. *p* < 0.05 was considered as statistically significant. All the statistical analyses were performed using the SPSS software package (version 24) (SPSS Incorporation, Chicago, Illinois, USA) and the GraphPad Prism version 8 (GraphPad Software Incorporation, San Diego, California, USA).

## Results

### Characteristics of Donors and Recipients

The demographic data of donors are given in Table 1. Among them, 5 (33.33%) donors had hypertension and 2 (13.33%) donors had diabetes mellitus. A total of 10 (66.67%) donors died of cerebral hemorrhage and 5 (33.33%) donors had experienced cardiac arrest. The average length of intensive care unit (ICU) stay was 4.53 days. Terminal serum creatinine was 121.42 μmol/l and the urine output per hour was about 164.50 ml/h. All 15 donors had uneventful intraoperative courses.

**Table 1:** Donor characteristics.

**Donor characteristics**	**Mean ± SD or *n* (%)**	**Range**
Age (years)	50.67 ±10.90	19–66
Gender (%)		
Male	9 (60.00)	
Female	6 (40.00)	
BMI (kg/m^2^)	23.29 ± 2.72	17.58–29.30
Hypertension (%)		
Yes	5 (33.33)	
No	10 (66.67)	
Diabetes (%)		
Yes	2 (13.33)	
No	13 (86.67)	
Cardiac arrest (%)		
Yes	5 (33.33)	
No	10 (66.67)	
Donor cause of death (%)		
Cerebral hemorrhage	10 (66.67)	
Accident	4 (26.67)	
Anoxia	1 (6.66)	
Length of stay in ICU (days)	4.53 ± 2.82	2.50–11.00
Terminal Scr (umol/L)	121.42 ± 73.09	47.25–284.50
Urine output (ml/h)	164.50 ± 53.75	109.25–300.50

*BMI, body mass index; ICU, intensive care unit; Scr, serum creatinine*.

The characteristics of recipients are shown in Table 2. Two recipients had received peritoneal dialysis and the others had received hemodialysis. The dialysis duration was 54.07 months and 46.80 months for the RP group and the AP group, respectively. All the patients received primary kidney transplantation and standard triad immunosuppressive regimen, with no difference in induction therapy and HLA-mismatch (HLA-MM) between the two groups (RP vs. AP, 4.27 vs. 3.93, *p* = 0.33).

**Table 2:** Recipient characteristics.

**Characteristics**	**RP group**	**AP group**	***P***
Number, n	15	15	NS
Sex, M/F	8/7	11/4	0.45
Age, years	45.33 ± 11.34	39.73 ± 8.58	0.14
BMI, kg/m^2^	20.60 ± 1.77	20.25 ± 3.24	0.72
Type of dialysis (HD/PD)	14/1	14/1	NS
Dialysis duration, months	54.07 ± 32.20	46.80 ± 24.89	0.50
Recipient retransplant	0	0	NS
Mean PRA, %	0	0	NS
HLA-MM (mean ± SD)	4.27 ± 1.03	3.93 ± 0.80	0.33
Lymphocytotoxicity test, %	2	2	NS
Induction agent, (%)			0.14
rATG	6 (40.0)	10 (66.7)	
Basiliximab	9 (60.0)	5 (33.3)	
Immunosuppression, n (%)			NS
CsA + MMF + S	0 (0)	1 (6.7)	
FK + MMF + S	15 (100)	14 (93.3)	

*RP, retrograde perfusion; AP, antegrade perfusion; M, male; F, female; BMI, body mass index; HD, hemodialysis; PD, peritoneal dialysis; Scr, serum creatinine; eGFR, estimated glomerular filtration rate; CysC, cystatin C; BUN, blood urea nitrogen; PRA, panel reactive antibody; HLA-MM, human leukocyte antigen-mismatch; rATG, rabbit antithymocyte globulin; CsA, cyclosporine A; FK, tacrolimus; MMF, mycophenolate mofetil; S, steroid; NS, no statistically significant*.

### Parameters of Perfusion

**Figure 2:**
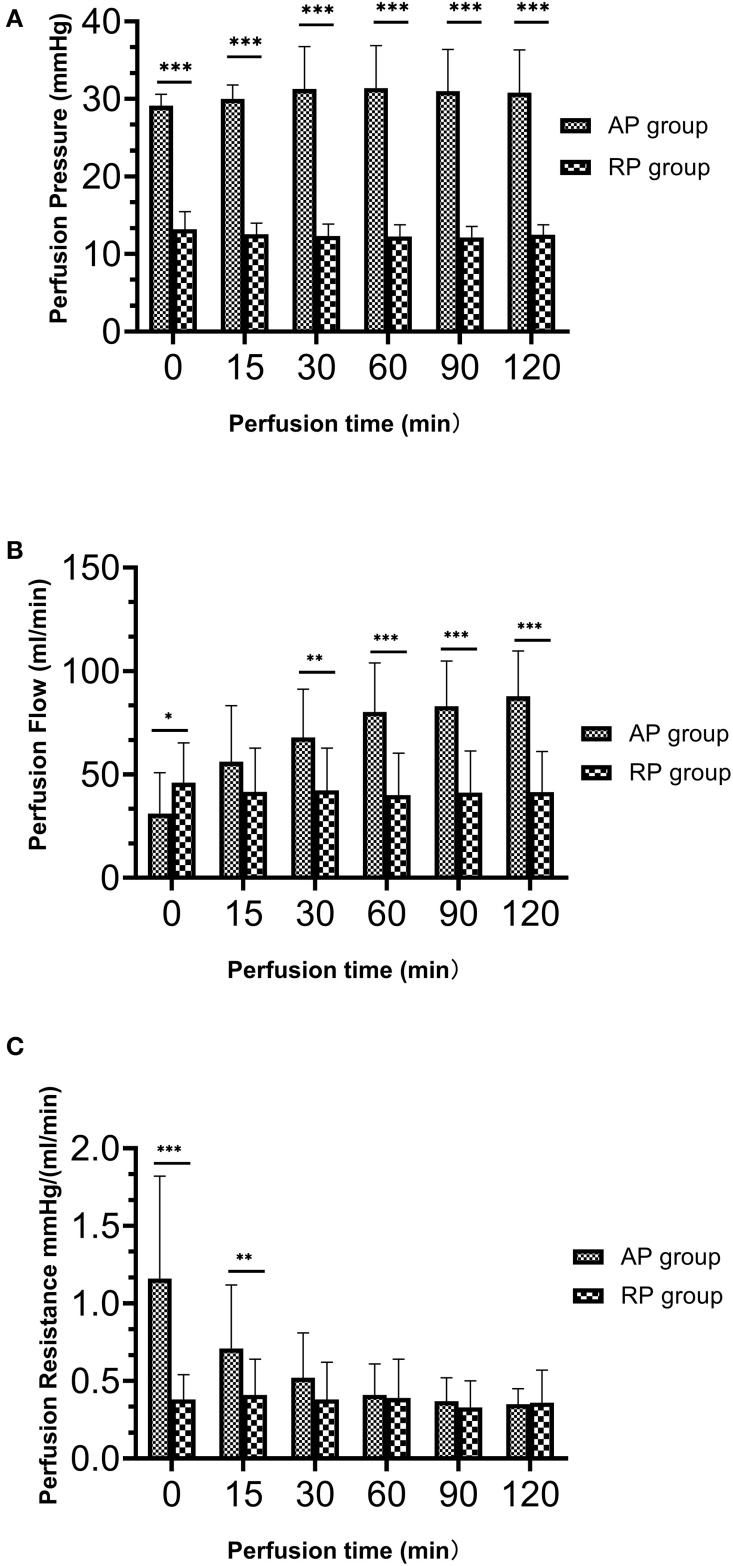
The dynamic perfusion parameters during hypothermic machine perfusion between both the groups. **(A)** Perfusion pressure (mm Hg); **(B)** Perfusion flow (ml/min); **(C)** Perfusion resistance [mm Hg/(ml/min)] (*p = 0.05 between two groups; **p > 0.01 but < 0.05 between two groups; ***p < 0.01 between two groups.

Details of the perfusion parameters are shown in Figure 2 and Table 3. There was no difference in WIT, CIT, and PT in both groups. Due to the artificial setting, the AP group had significantly higher initial and maintenance perfusion pressure than that in the RP group (*p* < 0.01). At the beginning of perfusion (PT = 10 min), the RP group had higher perfusion flow than the AP group (RP vs. AP, 45.93 vs. 31.07, *p* = 0.05), but the RP group had significantly lower terminal perfusion flow (RP vs. AP, 42.00 vs. 90.67 ml/min, *p* < 0.01). The RP group had lower initial perfusion resistance [RP vs. AP, 0.36 vs. 1.37 mm Hg/(ml/min) at PT 10 min, *p* < 0.01], while no difference was found between the two groups in terminal perfusion resistance [RP vs. AP, 0.30 vs. 0.32 mm Hg/(ml/min), *p* = 0.59]. During the perfusion, the resistance in the RP group was relatively stable (Figure 2C, Supplementary Table S1, Supplementary Figure S1).

**Table 3:** The parameters of perfusion in two groups.

**Variables**	**RP group**	**AP group**	***P***
Number (n)	15	15	NS
Warm ischemia time, min	2.79 ± 0.62	2.79 ± 0.62	NS
Cold ischemia time, h	10.71 ± 3.99	9.30 ± 3.77	0.33
Perfusion time, h	6.64 ± 3.87	5.25 ± 3.95	0.34
Initial perfusion pressure, mmHg	13.20 ± 2.27	29.13 ± 1.46	<0.01
Initial perfusion flow, ml/min	45.93 ± 19.37	31.07 ± 19.78	0.05
Initial perfusion resistance, mmHg/(ml/min)	0.38 ± 0.16	1.16 ± 0.66	<0.01
Perfusion pressure (2h), mmHg	12.47 ± 1.30	30.80 ± 5.53	<0.01
Perfusion flow (2h), ml/min	41.53 ± 19.62	87.80 ± 21.83	<0.01
Perfusion resistance (2h), mmHg/(ml/min)	0.36 ± 0.21	0.35 ± 0.10	0.84
Terminal perfusion pressure, mmHg	12.40 ± 1.50	30.40 ± 5.65	<0.01
Terminal perfusion flow, ml/min	42.07 ± 21.23	90.53 ± 24.12	<0.01
Terminal perfusion resistance, mmHg/(ml/min)	0.30 ± 0.16	0.32 ± 0.10	0.59

*RP, retrograde perfusion; AP, antegrade perfusion; NS, no statistically significant*.

### Transplantation Outcomes

**Figure 3:**
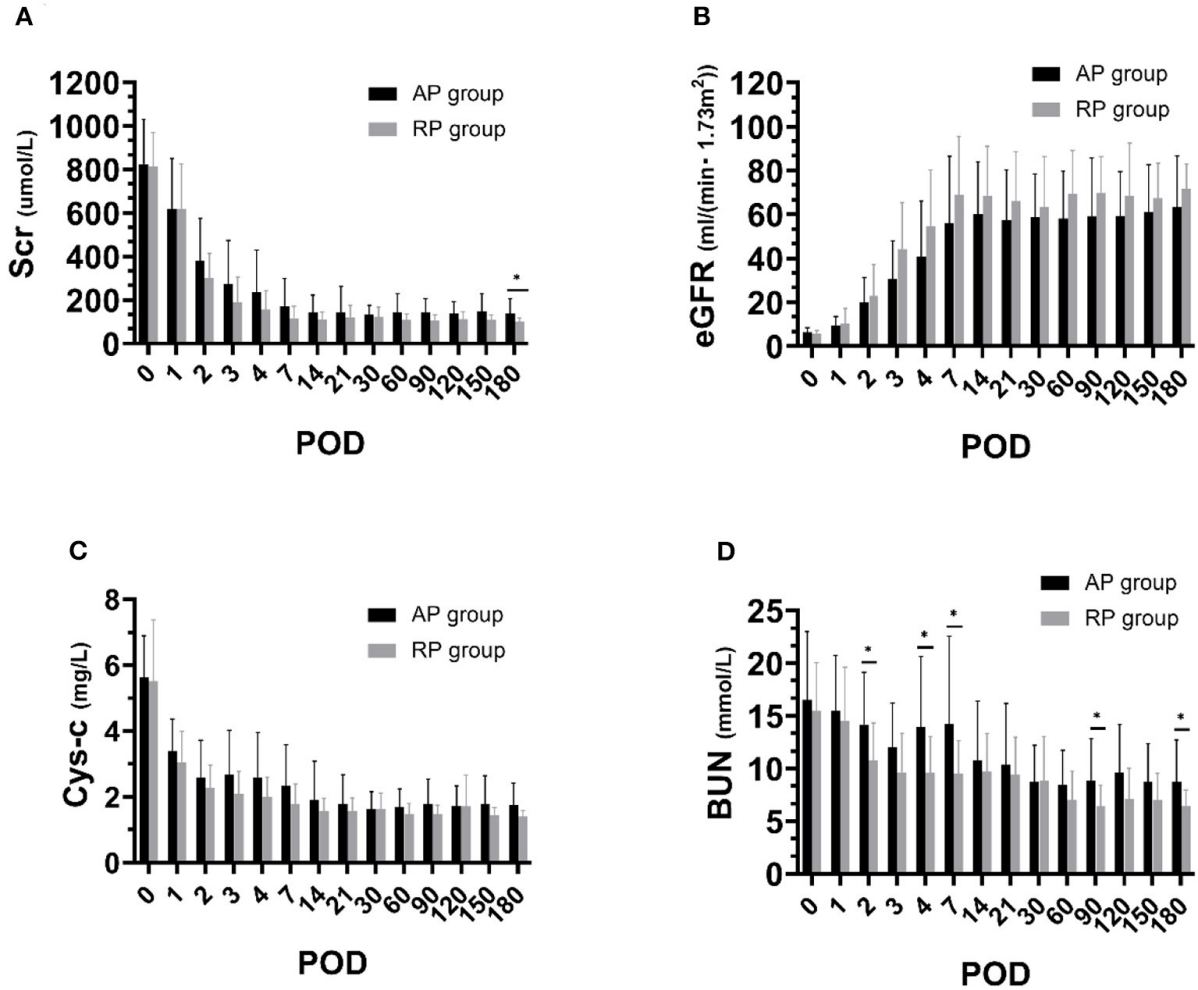
Renal function during postoperative 6 months between both the groups. **(A)** Serum creatinine (Scr); **(B)** Blood urea nitrogen (BUN); **(C)** Estimated glomerular filtration rate (eGFR); **(D)** Cystatin C (CysC) (*p < 0.05 between two groups).

**Figure 4:**
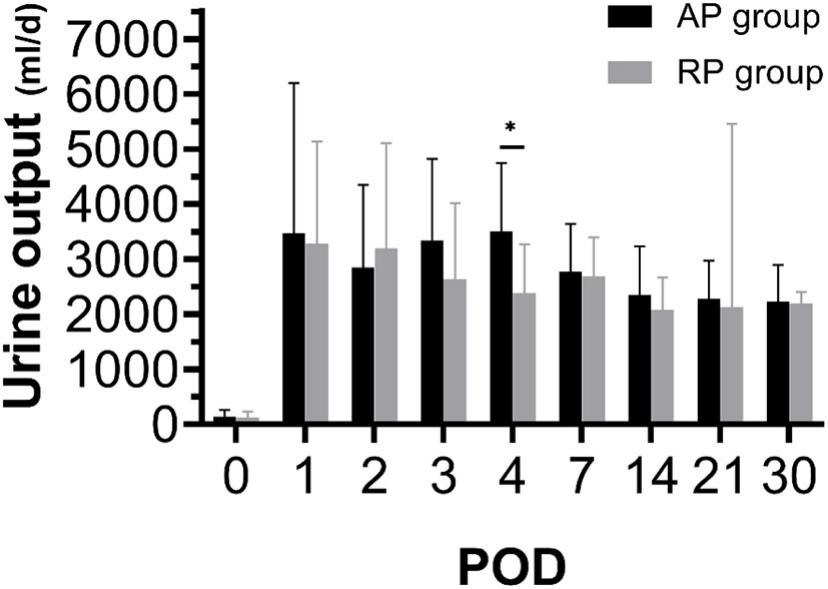
Twenty-four hours urine output during postoperative 1 month between both the groups (*p < 0.05 between two groups).

All the patients were followed up for 6 months. Postoperative information is given in Table 4. There was no primary non-function (PNF) in both groups. Three cases had DGF in the RP group and 2 cases had DGF in the AP group. DGF in the RP group lasted for 1 to 2 days, with 1 or 2 sessions of dialysis. Similarly, DGF occurred in the AP group lasted 1 or 3 days (Supplementary Table S2). As indicated in Figures 3, 4, Table 4, Supplementary Figure S2 and Supplementary Table S3, we found that patients who received RP perfused kidney had comparable urine output, Scr, CysC, BUN, and eGFR at any time point in the first month to those receiving AP perfused grafts. In postoperative 6 months, we found that the RP group had lower Scr (RP vs. AP, 102.20 vs. 138.67, *p* = 0.05) and BUN (RP vs. AP, 6.44 vs. 8.71, *p* = 0.05) than the AP group. There was no statistically significant difference between eGFR and CysC in POD 180 (*p* > 0.05). One clinically suspected acute rejection episode occurred in the RP group and three clinically suspected acute rejection episodes occurred in the AP group and all received methylprednisolone pulse therapy and recovered. There was no significant difference in the length of hospital stay between the two groups (RP vs. AP, 21.87 vs. 19.73, *p* = 0.39). There was no surgical-related complication such as wound infection, urinary leakage, or ureter stricture during the follow-up.

**Table 4:** The clinical outcome of kidney transplantation in both groups.

**Variables**	**RP group**	**AP group**	***P***
Number	15	15	NS
DGF, (%)	3 (20.00)	2 (13.33)	NS
PNF, (%)	0 (0)	0 (0)	NS
Suspected acute rejection, (%)	1 (6.67)	3 (20.00)	0.60
Wound infection, (%)	0	0	NS
Urinary fistula, (%)	0	0	NS
Hospital stays, days	21.87 ± 8.13	19.73 ± 4.80	0.39
Urine output at Pod 30, ml	2203.33 ± 205.69	2230.00 ± 671.83	0.88
Scr at Pod 30, umol/L	120.47 ± 44.54	131.07 ± 44.53	0.60
eGFR at Pod 30, ml/(min 1.73m^2^)	63.57 ± 22.84	58.91 ± 19.63	0.55
Cys-c at Pod 30, mg/L	1.63 ± 0.48	1.62 ± 0.53	0.97
BUN at Pod 30, mmol/L	8.82 ± 4.22	8.72 ± 3.47	0.95
Scr at Pod 60, umol/L	108.00 ± 28.27	143.13 ± 85.68	0.14
eGFR at Pod 60, ml/(min 1.73m^2^)	69.21 ± 19.98	58.08 ± 21.68	0.18
Cys-c at Pod 60, mg/L	1.47 ± 0.33	1.67 ± 0.56	0.22
BUN at Pod 60, mmol/L	7.04 ± 2.73	8.47 ± 3.26	0.20
Scr at Pod 90, umol/L	107.47 ± 24.85	141.20 ± 64.55	0.07
eGFR at Pod 90, ml/(min 1.73m^2^)	69.65 ± 16.71	59.06 ± 26.68	0.25
Cys-c at Pod 90, mg/L	1.48 ± 0.27	1.78 ± 0.76	0.16
BUN at Pod 90, mmol/L	6.40 ± 2.02	8.81 ± 4.01	0.05
Scr at Pod 180, umol/L	102.20 ± 16.21	138.67 ± 66.73	0.05
eGFR at Pod 180, ml/(min 1.73m^2^)	71.60 ± 11.43	63.57 ± 23.04	0.28
Cys-c at Pod 180, mg/L	1.40 ± 0.18	1.75 ± 0.67	0.06
BUN at Pod 180, mmol/L	6.44 ± 1.51	8.71 ± 3.99	0.05

*POD, postoperative day; Scr, serum creatinine; eGFR, estimated glomerular filtration rate; CysC, cystatin C; BUN, blood urea nitrogen; DGF, delayed graft function; PNF, primary non-function; NS, no statistically significant*.

At 1 week, all the allografts received the evaluation of ultrasound and there was no difference in renal, segmental, interlobar, and arcuate arterial resistance index (Supplementary Table S4). In the RP group, we divided the RP perfused grafts into two subgroups according to the perfusion resistance at 2 h (group 1, perfusion resistance < 0.4; group 2, perfusion resistance ≥ 0.4). In subgroup analysis, no difference was found in the arterial resistance index (Supplementary Table S5). Similarly, as shown in Supplementary Table S6, both the groups had a comparable renal function.

## Discussion

In this study, we first used RP machine perfusion for the preservation of kidneys from a deceased donor and found that kidneys receiving RP had a comparable incidence of DGF, urine output to the AP perfused allograft. Interestingly, although both the groups had comparable eGFR, we found that allografts perfused by RP had lower Scr and BUN than those receiving AP perfusion.

In organ procurement, we may come across renal artery injury, anatomical variation, and malformations of the arteries. These kidneys might not be well perfused through traditional arterial-to-venous perfusion (AP), which might increase the discard rate (15). Each renal segment was supplied by a segmental artery as an end-artery. In the back-table preparation, for kidneys with multiple arteries, effective perfusion of the whole kidney requires separate cannulation and flushing of each renal artery, which was time-consuming and laborious and the perfusion was not satisfying. Renal veins had greater diameters, less variation than renal arteries, and no venous valves in the renal venous system (16). Most importantly, there was extensive communication between segments on the venous side. In view of the anatomical difference between arteries and veins, it seemed to be possible to perfuse from veins to arteries and evidence from the animal study has demonstrated that renal perfusion could be carried out by retrograde blood flow from the efferent artery to the afferent artery (17).

Wilhelm et al. (18) had used the RP technique *in-situ* perfusion of dog model for the first time. Until the late 1980s, Rolles et al. (19) had carried out a clinical study on retrograde oxygen perfusion of renal grafts. Although this technique had not been further applied in transplantation due to the improvement of preservation solutions, it provided us with the feasibility of RP. An animal study found that 24 h RP of rabbit kidneys revealed good morphological changes (12). To further verify its feasibility and safety, Han et al. (11) conducted a porcine renal autotransplantation and found no difference in the renal function between the AP and the RP groups at day 7. Of note, Han et al. (15) compared the AP and RP in kidneys with damaged or variant arteries and found comparable graft survival at 1, 3, and 5 years. In another case series study, Hobeika et al. (20) also reported no difference in eGFR between the RP and the AP groups. These studies have indicated that RP of the kidney is feasible and safe.

In this study, we found no difference in the incidence of DGF, urine output, and renal function in the first month between the AP and RP groups. However, we found that the RP group had lower Scr and BUN than the AP group at 6 months, indicating that retrograde machine perfusion of allograft was not inferior to the conventional technique. Experience from lung transplant had indicated that retrograde flush could remove residual microthrombi after antegrade flush (21) and microthrombi were often found in kidneys from a deceased donor and these grafts may have a higher incidence of DGF and inferior early function (22). These facts raised the hypothesis that the RP technique might also help to remove microthrombi in the kidneys and improve organ perfusion and function preservation, while further evidence is required to verify it.

Initially, we set the perfusion pressure for RP at 15 mm Hg and found that the perfusion pump worked well and the solution could go smoothly into all the kidneys. The normal pressure in the renal vein is 10 mm Hg and higher venous pressures have been associated with impaired renal function (23). Thus, we planned to gradually lower the perfusion pressure down to 10 mm Hg. To the best of our knowledge, the lowest pressure for the perfusion pump to work in RP was 12 mm Hg. Therefore, we gradually decreased the pressure to 12 mm Hg every 10 min with 1 mm Hg lower. For some cases, we maintained the pressure at 15 mm Hg because when we lowered the pressure by 1 mm Hg, the perfusion pump failed to work. Interestingly, although the perfusion pressure and flow were much higher in the AP group, the perfusion resistance of the AP group gradually decreased and become very close to that of the RP group after 2 h. Of note, the perfusion resistance for most cases remained stable from the beginning to the end of perfusion in the RP group. Previous studies had indicated that perfusion resistance of allografts undergoing HMP (AP technique) was considered as a measure of organ quality (24) and the resistance often took a long time to obtain, usually more than 2 h. Therefore, our results suggested that retrograde machine perfusion might make a quicker assessment of kidney quality than the conventional perfusion technique.

In addition, most studies set the threshold of 0.4 mm Hg/ml/min for the perfusion resistance (AP technique) and found that resistance greater than 0.4 was associated with increased graft failure, even not being used for transplantation (25). In this study, we categorized the patients in the RP group according to the perfusion resistance at 2 h and found that the renal function in patients receiving kidneys with resistance <0.4 was similar to those patients receiving kidneys with resistance ≥0.4. Additionally, evidence from AP has shown that early renal Doppler ultrasound intrarenal resistive index was associated with detrimental pathological changes (26) and can help to predict long-term graft function (27). Our results found that kidneys with resistance < 0.4 had a comparable renal resistance index at Day 7, suggesting that resistance of 0.4 obtained from the RP technique is not a proper cutoff value for organ quality assessment and more evidence is required to find a resistance threshold of clinical significance.

There are several limitations to this study. First, we reported our early experience of RP techniques and the included cases were limited, which may be underpowered to detect the difference between the AR and RP groups. Second, we only included kidneys from DBD donors, and how the RP work in kidneys from cardiac death donor or those with acute kidney injury remained unknown. In addition, after perfusion satisfied by cannulating the renal vein to LifePort, we usually removed the joint part of the renal vein before the operation, which may lead to a shorter renal graft vein and increase the difficulty of vascular anastomosis, especially for retransplant or obese patients, since it has not happened in this study. Finally, we did not obtain biopsy data that whether RP could preserve the microstructure efficiently is still at issue. Finally, due to the short follow-up period, the long-term effect of RP is not clear. Therefore, a prospective trial with a greater number of participants and long-term follow-up is necessary to prove the equivalence or superiority of retrograde machine perfusion in kidney preservation.

## Conclusion

Machine perfusion with RP technique is effective and safe in preserving kidneys from deceased donors.

## Data Availability Statement

The original contributions presented in the study are included in the article/Supplementary Material, further inquiries can be directed to the corresponding author/s.

## Ethics Statement

The studies involving human participants were reviewed and approved by the Ethics Committee of West China Hospital of Sichuan University. The patients/participants provided their written informed consent to participate in this study.

## Author Contributions

JZ conceived and designed the study and prepared the first draft of the manuscript. ZJ performed the statistical analysis. TL analyzed the data. TS interpreted the data and revised the manuscript. All authors approved the final manuscript and contributed intellectually important content of the manuscript.

## Funding

This study was supported by the Natural Science Foundation of China (Grant Nos. 81870513, 81470980, and 81600584); 1.3.5 Project for Disciplines of Excellence, West China Hospital, Sichuan University (Grant No. ZY2016104); the Youth Researcher Funding of Sichuan University (Grant No. 2017SCU11042); the Research Funding of Sichuan Health and Family Planning Commission (Grant Nos. 17PJ159, 18PJ434, and 18PJ453).

## Conflict of Interest

The authors declare that the research was conducted in the absence of any commercial or financial relationships that could be construed as a potential conflict of interest.

## Publisher's Note

All claims expressed in this article are solely those of the authors and do not necessarily represent those of their affiliated organizations, or those of the publisher, the editors and the reviewers. Any product that may be evaluated in this article, or claim that may be made by its manufacturer, is not guaranteed or endorsed by the publisher.
