# Supplementary material for: Machine Retrograde Perfusion of Deceased Donor Kidneys: A Prospective Study
Source: Front Med (Lausanne). 2021 Dec 17;8:785953. doi: 10.3389/fmed.2021.785953 (PMC8718700; doi:10.3389/fmed.2021.785953)
Supplement: Supplementary file 1 [file Data_Sheet_1.docx]

**Table S1** The parameters of perfusion in two groups.

| **Time**  **(min)** | **Perfusion Pressure** **(mmHg)** | | | **Perfusion Flow (ml/min)** | | | **Perfusion Resistance (mmHg/(ml/min))** | | |
| --- | --- | --- | --- | --- | --- | --- | --- | --- | --- |
|  | RP | AP | P | RP | AP | P | RP | AP | P |
| PT 0 | 13.20 ± 2.27 | 29.13 ± 1.46 | <0.01 | 45.93 ± 19.37 | 31.07 ± 19.78 | 0.05 | 0.38± 0.16 | 1.16± 0.66 | <0.01 |
| PT 15 | 12.53 ± 1.46 | 30.00 ± 1.81 | <0.01 | 41.60 ± 21.12 | 56.13 ± 27.12 | 0.11 | 0.41 ± 0.23 | 0.71 ± 0.41 | 0.02 |
| PT 30 | 12.33 ± 1.54 | 31.27 ± 5.47 | <0.01 | 42.33 ± 20.35 | 67.87 ± 23.36 | 0.03 | 0.38 ± 0.24 | 0.52± 0.29 | 0.16 |
| PT 60 | 12.27 ± 1.49 | 31.40 ± 5.49 | <0.01 | 39.93 ± 20.36 | 80.27 ± 23.69 | <0.01 | 0.39 ± 0.25 | 0.41 ± 0.20 | 0.80 |
| PT 90 | 12.13 ± 1.41 | 31.00 ± 5.37 | <0.01 | 41.20 ± 20.08 | 83.00 ± 21.87 | <0.01 | 0.33 ± 0.17 | 0.37 ± 0.15 | 0.56 |
| PT 120 | 12.47 ± 1.30 | 30.80 ± 5.53 | <0.01 | 41.53 ± 19.62 | 87.80 ± 21.83 | <0.01 | 0.36 ± 0.21 | 0.35 ± 0.10 | 0.84 |
| PT end | 12.40 ± 1.50 | 30.40 ± 5.65 | <0.01 | 42.07 ± 21.23 | 90.53 ± 24.12 | <0.01 | 0.30 ± 0.16 | 0.32 ± 0.10 | 0.59 |

RP, retrograde perfusion; AP, antegrade perfusion; PT, perfusion time; min, minute.

**Table S2** The observation data for the DGF in the study.

| **Recipient** | **Age** | **Sex** | **Perfusion** | **Duration of DGF (day)** | **Times of dialysis** | **POD 30 Scr (umol/L)** | **POD 30 eGFR**  **(ml/(min・1.73m^2^))** |
| --- | --- | --- | --- | --- | --- | --- | --- |
| 1 | 40 | F | RP | 1 | 1 | 254 | 26.16 |
| 2 | 26 | M | AP | 3 | 2 | 137 | 45.89 |
| 3 | 48 | F | AP | 1 | 1 | 164 | 41.67 |
| 4 | 48 | M | RP | 2 | 2 | 123 | 44.79 |
| 5 | 30 | M | RP | 1 | 1 | 71 | 98.79 |

DGF, delayed graft function; POD, postoperative day; Scr, serum creatinine; eGFR, estimated glomerular filtration rate.

.

**Table S3** The changes of renal function within six months after transplantation.

| **Time (d)** | **Scr (umol/L)** | | | **eGFR** **(ml/(min・1.73m^2^))** | | | **Cys-c (mg/L)** | | | **BUN (mmol/L)** | | | **Urine output (ml/d)** | | |
| --- | --- | --- | --- | --- | --- | --- | --- | --- | --- | --- | --- | --- | --- | --- | --- |
|  | RP | AP | P | RP | AP | P | RP | AP | P | RP | AP | P | RP | AP | P |
| POD 0 | 812.53 ± 157.01 | 824.80 ± 206.00 | 0.86 | 5.72 ± 1.43 | 6.38 ± 2.01 | 0.31 | 5.52 ± 1.86 | 5.62 ± 1.27 | 0.86 | 15.45 ± 4.59 | 16.49 ± 6.51 | 0.62 | 125.67 ± 108.45 | 132.67 ± 129.24 | 0.92 |
| POD 1 | 619.27 ± 206.68 | 618.87 ± 231.31 | 0.99 | 10.29 ± 6.93 | 9.53 ± 4.02 | 0.72 | 3.03 ± 0.96 | 3.40 ± 0.96 | 0.75 | 14.46 ± 5.14 | 15.45 ± 5.30 | 0.61 | 3277.00 ± 1864.40 | 3470.47 ± 2733.60 | 0.82 |
| POD 2 | 301.07 ± 113.53 | 381.40 ± 194.12 | 0.18 | 23.05 ± 14.13 | 19.79 ± 11.36 | 0.49 | 2.28 ± 0.68 | 2.58 ± 1.14 | 0.38 | 10.73 ± 3.60 | 14.17 ± 4.95 | 0.04 | 3192.33 ± 1918.39 | 2848.67 ± 1501.17 | 0.59 |
| POD 3 | 190.07 ± 115.82 | 274.33 ± 199.25 | 0.17 | 44.26 ± 21.13 | 30.45 ± 17.52 | 0.06 | 2.08 ± 0.69 | 2.67 ± 1.35 | 0.15 | 9.65 ± 3.70 | 12.01 ± 4.20 | 0.11 | 2634.67 ± 1386.50 | 3339.33 ± 1485.77 | 0.19 |
| POD 4 | 155.73 ± 87.21 | 234.20 ± 195.26 | 0.18 | 54.75 ± 25.48 | 40.68 ± 25.43 | 0.14 | 1.99 ± 0.60 | 2.59 ± 1.36 | 0.13 | 9.57 ± 3.43 | 13.93 ± 6.69 | 0.03 | 2385.33 ± 886.94 | 3510.00 ± 1241.57 | 0.01 |
| POD 7 | 117.40 ± 54.04 | 171.27 ± 127.01 | 0.14 | 69.00 ± 26.56 | 56.05 ± 30.50 | 0.23 | 1.77 ± 0.62 | 2.33 ± 1.25 | 0.13 | 9.54 ± 3.09 | 14.23 ± 8.33 | 0.05 | 2686.67 ± 713.20 | 2776.00 ± 867.24 | 0.76 |
| POD 14 | 111.20 ± 32.83 | 141.07± 81.18 | 0.19 | 68.64 ± 22.50 | 59.92 ± 24.04 | 0.61 | 1.57 ± 0.39 | 1.91 ± 1.17 | 0.30 | 9.75 ± 3.58 | 10.77 ± 5.62 | 0.56 | 2086.67 ± 585.38 | 2345.33 ± 888.04 | 0.35 |
| POD 21 | 121.00 ± 54.88 | 142.53 ± 121.00 | 0.34 | 66.05 ± 22.50 | 57.59 ± 22.69 | 0.32 | 1.57 ± 0.40 | 1.79 ± 0.88 | 0.37 | 9.43 ± 3.53 | 10.35 ± 5.83 | 0.61 | 2126.67 ± 335.87 | 2276.67 ± 695.87 | 0.46 |
| POD 30 | 122.47 ± 44.54 | 131.07 ± 44.53 | 0.60 | 63.57 ± 22.84 | 58.91 ± 19.63 | 0.55 | 1.63 ± 0.48 | 1.62 ± 0.53 | 0.97 | 8.82 ± 4.22 | 8.72 ± 3.47 | 0.95 | 2203.33 ± 205.69 | 2230.00 ± 671.83 | 0.88 |
| POD 60 | 108.00 ± 28.27 | 143.13 ± 85.68 | 0.14 | 69.21 ± 19.98 | 58.08 ± 21.68 | 0.18 | 1.47 ± 0.33 | 1.68 ± 0.56 | 0.22 | 7.04 ± 2.73 | 8.47 ± 3.26 | 0.20 | ND | ND | ND |
| POD 90 | 107.47 ± 24.85 | 141.20 ± 64.55 | 0.07 | 69.65 ± 16.71 | 59.06 ± 26.68 | 0.25 | 1.48 ± 0.27 | 1.78 ± 0.76 | 0.16 | 6.40 ± 2.02 | 8.81 ± 4.01 | 0.05 | ND | ND | ND |
| POD 120 | 112.93 ± 33.79 | 138.57 ± 54.22 | 0.13 | 68.51 ± 23.95 | 59.12 ± 20.41 | 0.31 | 1.71 ± 0.95 | 1.72 ± 0.61 | 0.98 | 7.10 ± 2.92 | 9.59 ± 4.58 | 0.09 | ND | ND | ND |
| POD 150 | 108.40 ± 22.87 | 147.57 ± 81.17 | 0.08 | 67.33 ± 15.31399 | 61.23 ± 21.52 | 0.44 | 1.44 ± 0.23 | 1.77 ± 0.87 | 0.17 | 7.01 ± 2.52 | 8.75 ± 3.59 | 0.14 | ND | ND | ND |
| POD 180 | 102.20 ± 16.21 | 138.67 ± 66.73 | 0.05 | 71.60 ± 11.43 | 63.57 ± 23.04 | 0.28 | 1.40 ± 0.18 | 1.75 ± 0.67 | 0.06 | 6.44 ± 1.51 | 8.71 ± 3.99 | 0.05 | ND | ND | ND |

d, day; RP, retrograde perfusion; AP, antegrade perfusion; Scr, serum creatinine; eGFR, estimated glomerular filtration rate; Cys-c, cystatin c; BUN, blood urea nitrogen; POD, postoperative day; ND, no data.

**Table S4** The arterial resistance index within one week after transplantation in both groups.

| Arterial resistance index | RP group | AP group | P |
| --- | --- | --- | --- |
| Renal arterial | 0.71 ± 0.07 | 0.74 ± 0.07 | 0.26 |
| Segmental arterial | 0.68 ± 0.06 | 0.71 ± 0.08 | 0.38 |
| Interlobar arterial | 0.64 ± 0.06 | 0.67 ± 0.07 | 0.25 |
| Arcuate arterial | 0.64 ± 0.06 | 0.63 ± 0.07 | 0.73 |

RP, retrograde perfusion; AP, antegrade perfusion.

**Table S5** Arterial resistance index was stratified by perfusion resistance.

**RP group**

| Arterial resistance index | PR<0.4 | PR≥0.4 | P |
| --- | --- | --- | --- |
| Renal arterial | 0.70 ± 0.01 | 0.71 ± 0.07 | 0.80 |
| Segmental arterial | 0.66 ± 0.07 | 0.69 ± 0.07 | 0.50 |
| Interlobar arterial | 0.61 ± 0.02 | 0.65 ± 0.06 | 0.24 |
| Arcuate arterial | 0.64 ± 0.05 | 0.64 ± 0.06 | 0.92 |

**AP group**

| Arterial resistance index | PR<0.4 | PR≥0.4 | P |
| --- | --- | --- | --- |
| Renal arterial | 0.70 ± 0.05 | 0.74 ± 0.07 | 0.34 |
| Segmental arterial | 0.66 ± 0.06 | 0.72 ± 0.09 | 0.34 |
| Interlobar arterial | 0.65 ± 0.03 | 0.68 ± 0.08 | 0.62 |
| Arcuate arterial | 0.58± 0.06 | 0.64 ± 0.07 | 0.18 |

RP, retrograde perfusion; AP, antegrade perfusion; PR, perfusion resistance index.

**Table S6** Kidney function was stratified by perfusion resistance in RP group.

RP, retrograde perfusion; d, day; Scr, serum creatinine; eGFR, estimated glomerular filtration rate; Cys-c, cystatin c; BUN, blood urea nitrogen.

| **Time**  **(d)** | **Scr (umol/L)** | | | **eGFR (ml/(min・1.73m^2^))** | | | **Cys-c (mg/L)** | | | **BUN (mmol/L)** | | | |
| --- | --- | --- | --- | --- | --- | --- | --- | --- | --- | --- | --- | --- | --- |
|  | PR<0.4 | PR≥0.4 | P | PR<0.4 | PR≥0.4 | P | PR<0.4 | PR≥0.4 | P | PR<0.4 | PR≥0.4 | P |  |
| POD 0 | 818.55 ± 158.96 | 723.00 ± 116.23 | 0.36 | 5.40 ± 1.16 | 7.07 ± 2.01 | 0.08 | 6.03 ± 1.45 | 4.44 ± 2.75 | 0.19 | 16.80 ± 4.00 | 9.67 ± 1.91 | 0.01 |  |
| POD 1 | 619.82 ± 238.791 | 647.67 ± 92.31 | 0.85 | 11.04 ± 8.04 | 8.33 ± 1.41 | 0.58 | 3.11 ± 1.12 | 2.85 ± 0.12 | 0.70 | 14.40 ± 5.96 | 14.70 ± 2.77 | 0.94 |  |
| POD 2 | 282.91 ± 115.68 | 357.67 ± 127.48 | 0.35 | 25.12 ± 15.74 | 18.51 ± 7.95 | 0.50 | 2.28 ± 0.75 | 2.25 ± 0.64 | 0.95 | 10.19 ± 3.82 | 10.87 ± 1.17 | 0.77 |  |
| POD 3 | 179.55 ± 124.09 | 219.67 ± 120.93 | 0.63 | 47.84 ± 21.78 | 38.16 ± 20.09 | 0.50 | 2.14 ± 0. 78 | 1.92 ± 0.48 | 0.65 | 9.50 ± 4.12 | 8.93 ± 1.69 | 0.82 |  |
| POD 4 | 149.64 ± 93.70 | 168.00 ± 91.93 | 0.77 | 57.74 ± 25.90 | 52.73 ± 27.89 | 0.77 | 2.00 ± 0.68 | 1.85 ± 0.36 | 0.72 | 9.66 ± 3.87 | 8.27 ± 0.76 | 0.56 |  |
| POD 7 | 113.27 ± 57.80 | 130.00 ± 58.13 | 0.67 | 71.58 ± 26.56 | 67.49 ± 32.96 | 0.83 | 1.78 ± 0.70 | 1.64 ± 0.49 | 0.76 | 9.52 ± 3.60 | 9.53 ± 1.38 | 0.99 |  |
| POD 14 | 104.91 ± 34.12 | 121.00 ± 23.90 | 0.46 | 72.65 ± 22.67 | 64.84 ± 18.16 | 0.60 | 1.53 ± 0.43 | 1.59 ± 0.20 | 0.82 | 9.69 ± 4.20 | 9.67 ± 1.07 | 0.99 |  |
| POD 21 | 107.45 ± 30.30 | 113.33 ± 24.54 | 0.76 | 69.24 ± 19.14 | 71.01 ± 23.02 | 0.89 | 1.47 ± 0.27 | 1.53 ± 0.25 | 0.76 | 9.00 ± 2.97 | 8.20 ± 2.02 | 0.67 |  |
| POD 30 | 121.45 ± 46.77 | 105.00 ± 21.66 | 0.57 | 63.18 ± 21.66 | 76.87 ± 20.12 | 0.35 | 1.65 ± 0.53 | 1.42 ± 0.26 | 0.48 | 9.07 ± 4.81 | 7.27 ± 1.72 | 0.55 |  |
| POD 60 | 102.36 ± 19.24 | 102.33 ± 20.03 | 0.99 | 69.51 ± 15.07 | 88.47 ± 16.94 | 0.14 | 1.44 ± 0.27 | 1.33 ± 0.23 | 0.56 | 6.63 ± 2.20 | 6.37 ± 2.15 | 0.86 |  |
| POD 90 | 106.00 ± 24.95 | 98.33 ± 11.24 | 0.62 | 69.68 ± 12.74 | 86.36 ± 13.95 | 0.13 | 1.47 ± 0.26 | 1.41 ± 0.30 | 0.76 | 5.91 ± 1.42 | 6.43 ± 1.76 | 0.60 |  |
| POD 120 | 112.73 ± 37.63 | 107.00 ± 25.71 | 0.81 | 67.37 ± 24.40 | 87.11 ± 11.00 | 0.31 | 1.82 ± 1.09 | 1.34 ± 0.26 | 0.48 | 6.42 ± 2.78 | 8.93 ± 3.38 | 0.21 |  |
| POD 150 | 107.45 ± 26.12 | 107.67 ± 12.74 | 0.99 | 67.74 ± 15.78 | 75.81 ± 15.71 | 0.53 | 1.46 ± 0.24 | 1.40 ± 0.31 | 0.75 | 6.54 ± 1.84 | 8.50 ± 4.75 | 0.26 |  |
| POD 180 | 98.91 ± 17.00 | 106.67 ± 6.66 | 0.46 | 73.78 ± 8.81 | 73.98 ± 9.30 | 0.97 | 1.40 ± 0.19 | 1.38 ± 0.25 | 0.83 | 6.46 ± 1.49 | 5.87 ± 1.80 | 0.57 |  |
