# Supplementary material for: Machine Retrograde Perfusion of Deceased Donor Kidneys: A Prospective Study
Source: Front Med (Lausanne). 2021 Dec 17;8:785953. doi: 10.3389/fmed.2021.785953 (PMC8718700; doi:10.3389/fmed.2021.785953)
Supplement: Supplementary file 2 [file Data_Sheet_2.docx]

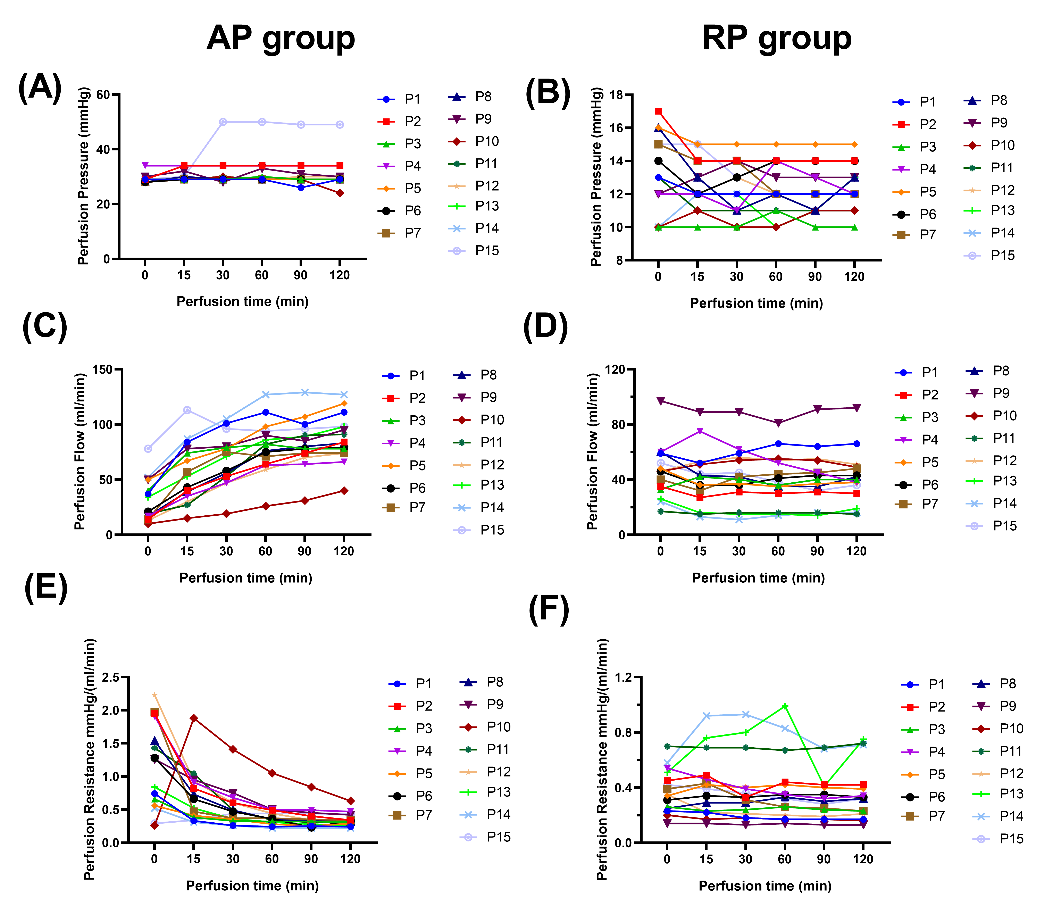


**Figure S1** The perfusion parameters of all kidney grafts. (A), Perfusion Pressure (mmHg) in antegrade perfusion group; (B), Perfusion Pressure (mmHg) in retrograde perfusion group; (C), Perfusion Flow (ml/min) in antegrade perfusion group; (D), Perfusion Flow (ml/min) in retrograde perfusion group; (E), Perfusion Resistance (mmHg/ml/min) in antegrade perfusion group; (F), Perfusion Resistance (mmHg/ml/min) in retrograde perfusion group.


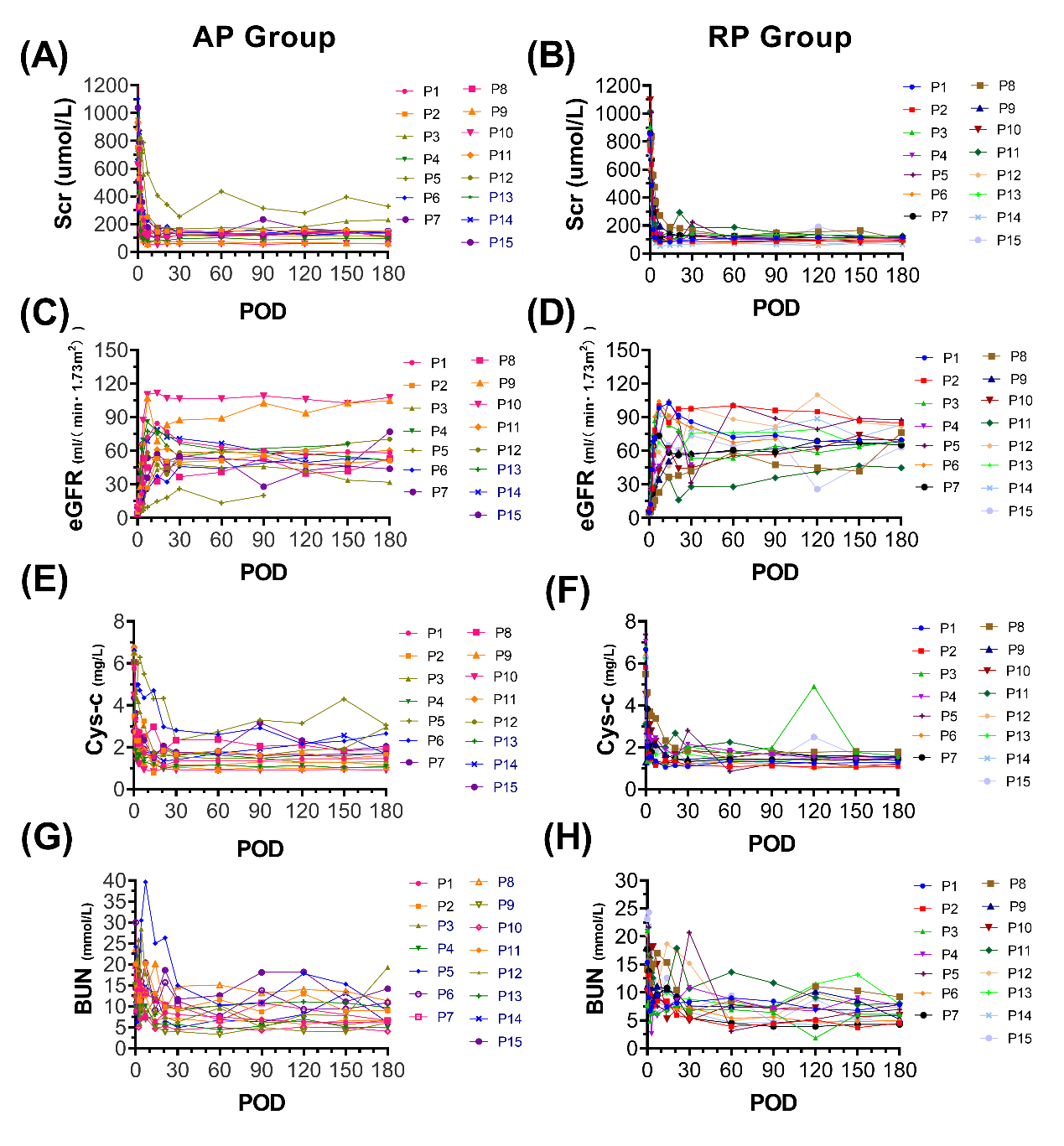


**Figure S2** Postoperative Kidney function of all recipients. (A), serum creatinine (Scr) in antegrade perfusion group; (B), serum creatinine (Scr) in retrograde perfusion group; (C), estimated glomerular filtration rate (eGFR) in antegrade perfusion group; (D), estimated glomerular filtration rate (eGFR) in retrograde perfusion group; (E), cystatin c (Cys-c) in antegrade perfusion group; (F), cystatin c (Cys-c) in retrograde perfusion group; (G), Twenty-four hours urine output in antegrade perfusion group; (H), Twenty-four hours urine output in retrograde perfusion group.
